# Emerging interplays between poxviruses and autophagy

**DOI:** 10.3389/fcimb.2025.1662511

**Published:** 2025-08-27

**Authors:** Yongge Li, Xu Miao, Rui Jia, Ruikang Liu

**Affiliations:** ^1^ Department of Genetics and Cell Biology, College of Life Sciences, Nankai University, Tianjin, China; ^2^ Key Laboratory of Molecular Microbiology and Technology, Ministry of Education, College of Life Sciences, Nankai University, Tianjin, China; ^3^ Department of Cell Biology, School of Basic Medical Sciences, Cheeloo College of Medicine, Shandong University, Jinan, Shandong, China; ^4^ Department of Pathogenic Biology, School of Basic Medical Sciences, Cheeloo College of Medicine, Shandong University, Jinan, Shandong, China

**Keywords:** poxvirus, autophagy, lysosome, virus-host interaction, oncolytic virus, monkeypox virus

## Abstract

Poxviruses are large double-stranded DNA viruses that replicate exclusively in the cytoplasm. Their life cycle is closely associated with various membrane-related cellular processes. This review summarizes current findings on the complex interplay between poxviruses and autophagy, as well as the endo-lysosomal network. However, due to the large diversity of poxvirus species and the limited number of relevant studies, it remains challenging to draw definitive conclusions regarding the bidirectional regulatory relationship between poxviruses and the autophagy-lysosome system. In addition, poxviruses can serve as a promising platform for oncolytic virus development. Furthermore, we also highlight recent studies leveraging autophagy modulation to enhance the therapeutic efficacy of oncolytic poxviruses. Thus, elucidation of the interplay between poxviruses and autophagy-lysosome pathway will not only advance the understanding of virology and cell biology, but also facilitate the engineering of oncolytic poxviruses as innovative tools for cancer therapy.

## 1 Introduction

Poxviruses have caused severe infectious diseases in humans. Variola virus (VARV), a member of the *Orthopoxvirus* genus in the *Poxviridae* family, causes smallpox, a devastating disease in humans until its eradication by vaccination in the 1980s (Bryant and Shulman, 2025). In recent years, monkeypox virus (MPXV), another Orthopoxvirus, has emerged as a global health threat. The World Health Organization (WHO) declared mpox (the human disease caused by MPXV) a Public Health Emergency of International Concern in 2022 and again in 2024 (Bryant and Shulman, 2025). Furthermore, certain species of poxviruses have been developed as invaluable tools in biomedical research, serving as gene delivery vehicles, vaccine vectors and oncolytic viruses (Evans et al., 1988; Breitbach et al., 2011; Liu et al., 2025).

Poxviruses are double-stranded DNA viruses with a broad host range (Oliveira et al., 2017). The life cycle of poxviruses is distinct from that of most conventional DNA viruses, which replicate in nuclei. Poxviruses encode their own DNA replication and transcription machineries, enabling them to complete the life cycle exclusively in the cytoplasm (Tolonen et al., 2001). As a result, viral genome, transcripts, proteins and other components are extensively exposed to the cytoplasmic environment. They are readily recognized as pathogen-associated molecular patterns (PAMPs), thereby triggering a range of host cellular responses (Lawler and Brady, 2020). Among them, autophagy has become an emerging point in the study of poxvirus-host interactions. This review distills current literature on the interactions between poxviruses and the autophagy-lysosome degradation pathway, and discusses the strategy to enhance oncolytic poxvirus efficacy via autophagy modulation.

## 2 The life cycle of poxviruses

**Figure 1:**
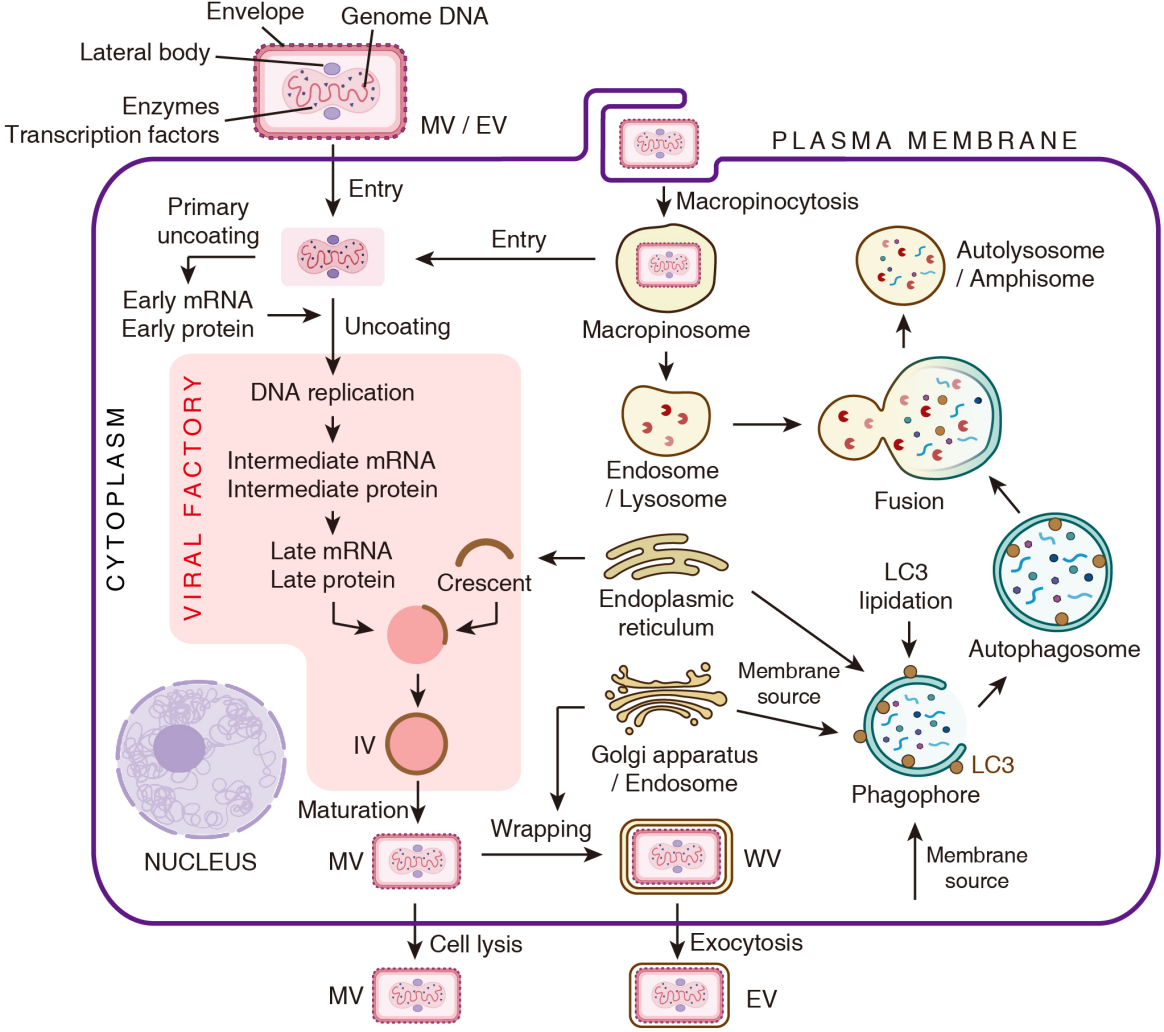
Schematic representation of the poxvirus life cycle and the autophagy pathway. The depiction of the viral life cycle is adapted from Chapter 16 of *Fields Virology* (Moss and Smith, 2021).

The quasi brick-shaped poxvirus virion consists of viral core composed of the genome and associated proteins, flanked by lateral bodies and encapsulated by a membrane envelope (**Figure 1**) (Peters, 1956; Cyrklaff et al., 2005). The entry of poxviruses into host cells is mediated by fusion of viral envelope with the plasma membrane or macropinosomes (Laliberte et al., 2011; Schmidt et al., 2011; Moss, 2016). Following entry, the poxvirus undergoes the primary uncoating process, during which early viral mRNAs are released from the core and translated into early proteins. Upon completed uncoating, genome replication and the expression of intermediate and late viral genes take place within a specialized cytoplasmic domain termed viral factory. These replication zones are typically positioned adjacent to the nucleus (**Figure 1**) (Tolonen et al., 2001; Moss and Smith, 2021).

The processes of virion assembly and viral membrane biogenesis are complex and highly orchestrated. Viral membrane assembly proteins (VMAPs) target to endoplasmic reticulum (ER), and generate crescent-shaped membrane fragments. These crescents assemble and encapsulate dense viroplasm to form spherical immature virions (IVs). The viroplasm comprises the viral genome, core protein precursors and the transcription machinery. Subsequently, IVs are transformed into brick-shaped mature virions (MVs), thereby acquiring infectivity. This maturation process involves IV surface restructuring and proteolysis of core proteins. Some MVs are wrapped by an additional double membrane derived from the Golgi apparatus or the early endosome, resulting in the formation of wrapped virions (WVs). These WVs can undergo exocytosis and lose one layer of membrane, ultimately generating double-membraned extracellular enveloped virions (EVs) (**Figure 1**) (Liu et al., 2014; Moss and Smith, 2021).

Notably, the membrane compartments involved in these processes, such as ER, endosomes, Golgi apparatus and plasma membrane, are crucial components of the autophagy pathway. This overlap suggests a potential and multifaceted interface between poxviruses and the autophagic process.

## 3 Autophagy-lysosome degradation pathway

Autophagy is a conserved catabolic process. Distinguished by the routes of cargo delivery to lysosomes, autophagy can be divided into macroautophagy, microautophagy and chaperone-mediated autophagy. Consequently, these three machineries are collectively referred to as the autophagy-lysosome degradation pathway (Kenney and Benarroch, 2015).

Macroautophagy (hereafter referred to as autophagy) is the most extensively studied subtype. It is characterized by the formation of a double-membraned autophagosome, which engulfs cytoplasmic components for degradation. Numerous reviews have comprehensively discussed this process in detail (Dikic and Elazar, 2018). Generally, the initiation of autophagy begins with the sequential activation of two kinase complexes, the ULK1 complex and class III phosphatidylinositol 3-kinase (PI3K) complex. PI3K catalyzes the production of phosphatidylinositol-3-phosphate (PI3P) on the ER to form the phagophore initiation site. Phagophore membrane expansion is supported by ATG9-mediated lipid transfer from multiple sources such as the Golgi apparatus, endosomes and the plasma membrane. Concurrently, the ATG8 conjugation system mediates the lipidation of ATG8 family proteins (e.g., LC3 and GABARAP), enabling their localization to the expanding phagophores. The matured autophagosome fuses with lysosomes and endosomes via SNARE proteins, forming autolysosomes and amphisomes, separately. Lysosomal enzymes then degrade the substrates, recycling nutrients back to the cytosol (**Figure 1**).

The interplay between autophagy and viruses is highly dynamic and virus-specific. This interaction resembles a “Holmes and Moriarty” scenario, with each adapting to outmaneuver the other. Generally, autophagy restricts viral infection by degrading virions or viral components. In contrast, many viruses have evolved strategies to inhibit autophagy flux, induce autophagic degradation of host antiviral factors, or even hijack autophagic machineries for their own replication (Jassey and Jackson, 2024; Münz et al., 2025). Despite these insights, whether and how poxviruses regulate autophagy, or are regulated by it, remains largely unknown.

## 4 Poxviruses modulate autophagy

**Figure 2:**
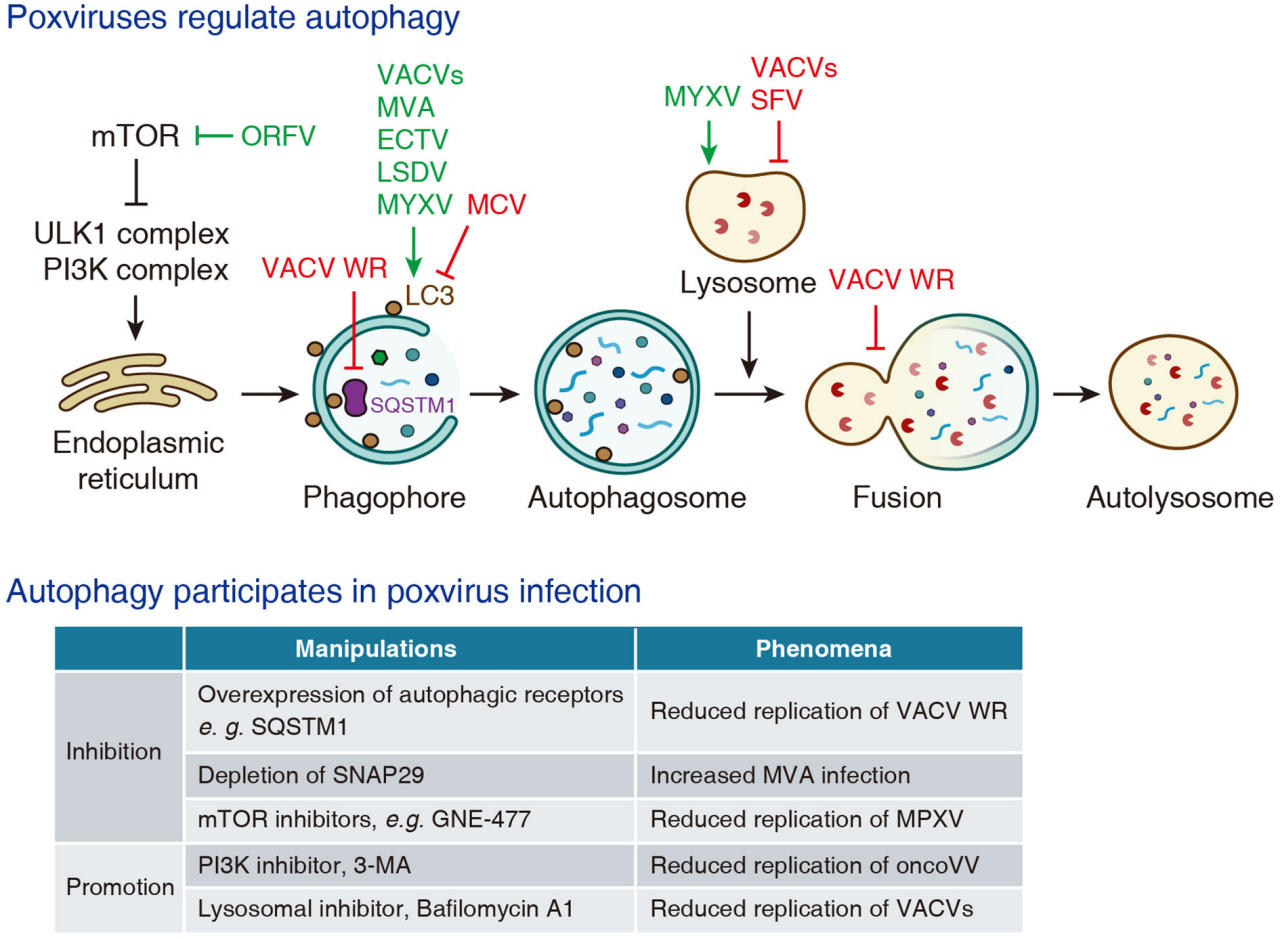
The interplay between poxviruses and autophagy.

Distinct poxviral strains have been shown to either activate or suppress autophagy activity, reflecting a context-dependent regulatory landscape. In the following, we will outline current evidences on how different species of poxviruses regulate autophagy (**Figure 2**).

### 4.1 Vaccinia virus

Vaccinia virus (VACV) belongs to the genus of *Poxviridae Orthopoxvirus*. It is now widely employed as a model strain in poxvirus research (Moss and Smith, 2021). Distinct strains of VACV exert diverse effects on autophagy.

The Western Reserve (WR) strain encodes two viral kinases, B1 and F10, that phosphorylate the autophagic receptor SQSTM1/p62 and promote its nuclear translocation. This relocalization impairs selective autophagy, potentially creating favorable cytoplasmic environment for viral replication (Krause et al., 2024). A recent preprint illustrates that WR virus A52 protein suppresses the fusion between autophagosomes and lysosomes by promoting proteasomal degradation of SNAP29, a component of SNARE complex mediating autophagosome-lysosome fusion (Niu et al., 2024). Several studies have reported that the infection of various VACV strains, such as WR (Ma et al., 2020), VG9 (Yang et al., 2019), VV-Onco oncolytic strain (Jia et al., 2016; Yu et al., 2024; Zhang et al., 2024) and Lister-dTK oncolytic strain (Whilding et al., 2013), induces LC3 lipidation and LC3-positive puncta formation. In another study, a modified VACV strain was shown to induce non-canonical conjugation of ATG12 to ATG3, and facilitating LC3 lipidation while paradoxically suppressing autophagosome formation (Moloughney et al., 2011).

### 4.2 Modified vaccinia virus Ankara

Modified vaccinia virus Ankara (MVA) was originally derived from the Ankara strain through more than 500 serial passages in primary chicken embryo fibroblasts (CEFs) (Mayr and Munz, 1964). This extensive passaging resulted in the accumulation of multiple genomic deletions and a loss of virulence in humans and most mammalian cells. Despite its attenuation, MVA retains strong immunogenicity against VARV and MPXV, which supported its early development as a first-generation vaccine for smallpox and mpox. MVA has been shown to increase LC3-II levels in BMDCs, promote the degradation of LC3 in Huh7 cells, and enhance autophagic flux in CMK leukemia cells (Thiele et al., 2015; Tappe et al., 2018). However, given the insufficiency of current evidences, it remains unclear whether MVA directly induces autophagy for successful replication or simply fails to suppress the innate immune response.

### 4.3 Orf virus

Orf virus (ORFV) is a zoonotic parapoxvirus belonging to *Poxviridae Parapoxvirus*. It primarily infects sheep and goats, causing contagious ecthyma, and can also infect humans through direct contact with infected animals (Haig, 2006). In primary ovine fetal turbinate (OFTu) cells and human nasopharyngeal carcinoma (NPC) cells, ORVF infection induces autophagy, which is characterized by the formation of autophagosomes and autolysosomes, increased LC3 lipidation and decreased SQSTM1 expression (Lan et al., 2016; Huang et al., 2022; Lv et al., 2023). Mechanistic studies have demonstrated that ORFV promotes autophagy by inhibiting the PI3K-AKT pathway and activating the ERK1/2 kinases, thereby suppressing mTOR kinase activity (Huang et al., 2022; Lv et al., 2023).

### 4.4 Ectromelia virus

Ectromelia virus (ECTV) is another member of the *Orthopoxvirus* genus, which causes mousepox, a highly contagious and often lethal disease in mice. Due to its high infectivity and pathogenicity, ECTV has become a well-established model for investigating poxvirus pathogenesis. Infection of fibroblast and macrophage cell lines with Moscow strain of ECTV (ECTV-MOS) markedly elevates LC3-II levels and increases the number of LC3-positive puncta (Martyniszyn et al., 2011, 2013c, 2013b; Gregorczyk et al., 2018). Furthermore, these *in cellulo* findings are corroborated by *in vivo* studies, that demonstrates the expression level of LC3-II, but not Beclin-1, is elevated in splenocytes from ECTV-MOS infected C57BL/6 and BALB/c mice (Martyniszyn et al., 2013a).

### 4.5 Other poxviruses

Molluscum contagiosum virus (MCV) is a human-specific poxvirus that belongs to the genus of *Poxviridae Molluscipoxvirus*. MCV infection causes molluscum contagiosum, a self-limiting skin disease marked by benign dome-shaped papules, mainly in children and the immunocompromised individuals (Chen et al., 2013). The MC159 protein of MCV is reported to reduce the formation of LC3-positive puncta, but without altering SQSTM1 levels (Schmotz et al., 2019).

Lumpy skin disease virus (LSDV), a member of *Poxviridae Capripoxvirus*, exhibits high host specificity and primarily infects cattle and buffaloes, causing lumpy skin disease (Whittle et al., 2023). In primary bovine embryonic fibroblasts (BEFs), LSDV infection leads to increased LC3-II levels and a higher number of LC3-positive puncta at 96 hours post-infection, implying that the virus promotes autophagy (Tan et al., 2023).

Myxoma virus (MYXV) is a member of *Poxviridae Leporipoxvirus*. MYXV naturally infects lagomorphs, causing the fatal disease myxomatosis, but results in no obvious pathology in humans or mice (Bartee et al., 2012). Supernatants from MYXV-infected human and murine B lymphocyte cell lines exhibit elevated LC3 protein levels at 48 hours post-infection, albeit as detected by ELISA (Yeşilaltay et al., 2024).

Collectively, these findings suggest that distinct poxvirus strains may exploit different mechanisms to interfere with autophagy, likely to optimize intracellular conditions for viral replication. LC3 lipidation and autophagosome formation appear to be preferred targets. The strain-specific differences in protein expression highlight a complex interface between poxviruses and the host autophagy machinery. For example, the absence of an MC159 homolog in VACV may contribute to the divergent autophagy responses induced by MCV and VACV (Biswas et al., 2018).

## 5 Autophagy regulates poxvirus infection

Autophagy is a well-known host defense mechanism, and is also involved in the replication of poxviruses (**Figure 2**). It has been reported that overexpression of autophagy receptors such as NDP52, SQSTM1 or TAX1BP1 significantly inhibits the replication of VACV WR strain (Krause et al., 2024). In a preprint study, depletion of SNAP29, a component of the SNARE complex that mediates autophagosome-lysosome fusion, is reported to enhance MVA infection (Niu et al., 2024). A preprint article shows that several mTOR inhibitors, such as GNE-477 and GDC-0980, reduce the replication of MPXV (Jeyachandran et al., 2025). Conversely, PI3K inhibitor 3-methyladenine (3-MA) is reported to reduce the replication of oncolytic vaccinia virus (oncoVV-AVL) (Yu et al., 2024), which implies that autophagy is required for efficient virus infection. In addition, in some cases autophagy seemingly has no effect on viral infection. One study reported that depletion of ATG3 did not significantly affect the replication of VACV in mouse embryonic fibroblasts (MEFs) (Moloughney et al., 2011).

Taken together, current studies on autophagy in poxvirus infection remain limited. Discrepancies across observations are likely attributable to differences in viral strains or experimental approaches. These variations may reflect the selective engagement of specific components of the autophagic machinery, wherein depletion of certain factors enhances viral replication, while others exert inhibitory effects. Additionally, manipulation of autophagy-related components may affect viral replication through autophagy-independent mechanisms, further contributing to the divergent results. For instance, class III PI3K has been reported to activate SGK1 and S6K (Byfield et al., 2005), and SQSTM1 is known to participate in NF-κB activation (Sanz et al., 1999).

## 6 Interaction between poxvirus and endo-lysosome system

The endo-lysosome system consists of a series of membrane organelles. Early endosomes function as the initial compartment for receiving and dispensing internalized cargo derived from various endocytic pathways. Late endosomes or multivesicular bodies (MVBs) serve as intermediates route to lysosomes. Lysosomes are terminal degradative compartments containing hydrolytic enzymes (Hu et al., 2015). Many viruses exploit this system for entry, replication or immune evasion, and poxviruses are no exception.

Macropinocytosis is a clathrin-independent, actin-driven form of endocytosis that enables the non-selective uptake of extracellular fluid and solutes into large vesicles termed macropinosomes (Tu et al., 2025). Studies using chemical inhibitors have demonstrated that macropinocytosis is indispensable in the entry of various poxviruses, *e.g.* VACV, ORFV and LSDV (Mercer and Helenius, 2008; Schmidt et al., 2011; Tang et al., 2023; Wang et al., 2024). Importantly, acidification of macropinosomes is also required for virus-host membrane fusion and productive viral entry (Mercer et al., 2010; Schmidt et al., 2011; Rizopoulos et al., 2015). Moreover, enveloped virions (EVs) show a stronger preference for macropinocytic uptake compared to mature virions (MVs), which is considered to utilize multiple redundant entry mechanisms (Sandgren et al., 2010).

Lysosomes, the terminal organelles of both the endo-lysosomal and autophagy-lysosomal pathways, have also been implicated with poxvirus infection (**Figure 2**). Early studies using VACV Lister strain, VACV Utrecht strain (previously classified in *Leporipoxvirus*), and Shope fibroma virus (SFV, belongs to the genus of *Leporipoxvirus*) demonstrate that poxvirus infection leads to lysosomal membrane destabilization and release of lysosomal enzymes into the cytoplasm (Allison and Sandelin, 1963; Ogier et al., 1974; Schümperli et al., 1978). In contrast, another report indicates that MYXV infection enhances lysosomal degradation of MHC-I molecules in baby green monkey kidney (BGMK) cells, and this effect is attenuated by pharmacological inhibitors of lysosomal function (Zúñiga et al., 1999).

On the other hand, inhibition of lysosomal acidification by the v-ATPase inhibitor Bafilomycin A1 has been shown to reduce the replication of VACV IHD-J, WR and oncoVV strains (Mercer et al., 2010; Schmidt et al., 2011; Niu et al., 2024). Considering autophagy has been reported to restrict poxvirus infection (Krause et al., 2024), and inhibition of lysosomal function would be expected to impair autophagic degradation and thereby enhance viral replication, the observed antiviral effect of Bafilomycin A1 presents a paradox. This discrepancy may reflect autophagy-independent effects of Bafilomycin A1 on other cellular pathways essential for efficient viral replication.

Nevertheless, these findings collectively suggest a bidirectional regulation between poxviruses and lysosomal function, involving both virus-induced lysosomal membrane perturbation and lysosome-dependent steps in the viral life cycle.

## 7 Autophagy modulation in oncolytic poxvirus engineering

Oncolytic viruses (OVs) selectively infect and kill tumor cells while sparing normal tissues, acting through both direct cell lysis and indirect immune activation (Shen and Nemunaitis, 2005; Chernichenko et al., 2013). OVs are genetically engineered to enhance tumor selectivity, restrict replication in normal cells, and express therapeutic transgenes (Kaufman et al., 2015). Among various OV platforms, poxviruses are particularly attractive due to their large genome, cytoplasmic replication and broad host range. Poxviruses with deletions in thymidine kinase (TK) or vaccinia growth factor (VGF) genes preferentially replicate in tumor cells characterized by high proliferation rates and elevated intracellular dNTP pools (Kaufman et al., 2015).

Multiple clinical trials have confirmed the anti-tumor activity and favorable safety profile of VACV-based OVs. In a phase III trial, intraperitoneal Olvimulogene nanivacirepvec (Olvi-Vec, also known as GL-ONC1) in platinum-resistant ovarian cancer leads to >50% response rate, with median progression-free and overall survival of 11.0 and 15.7 months, respectively (Holloway et al., 2023). In another phase I trial, administration of Olvi-Vec in combination with cisplatin and radiotherapy for advanced head and neck cancer resulted in a 1-year progression-free survival rate of 74.4% and an overall survival rate of 84.6% (Mell et al., 2017).

Although detailed mechanisms of poxvirus-autophagy interactions remain underexplored, autophagy modulation has already emerged as a promising strategy to enhance the efficacy of oncolytic poxviruses. A VACV-based oncolytic virus OVV-BECN1 is engineered by replacing viral TK gene with Beclin-1, a component of the class III PI3K complex that initiates autophagy (Lei et al., 2020; Xie et al., 2021). Infection with OVV-BECN1 robustly increases autophagy flux and exhibits greater cytotoxic activity in human leukemia, multiple myeloma and non-Hodgkin lymphoma cell lines (Lei et al., 2020; Xie et al., 2021). Mechanistic studies using pharmacologic inhibitors and siRNA-mediated gene silencing indicate that OVV-BECN1 induces autophagic cell death rather than apoptosis (Lei et al., 2020; Xie et al., 2021). In xenograft models of lymphoma and leukemia established by subcutaneous implantation of OCI-LY3 and K562 cells into BALB/c nude mice, OVV-BECN1 infection significantly suppresses tumor growth and prolongs survival rate (Lei et al., 2020; Xie et al., 2021).

In addition to direct genetic engineering using autophagy-related genes, pharmacological activators of autophagy have also been utilized to enhance the therapeutic efficacy of oncolytic poxviruses. For instance, combination therapy with rapamycin has been shown to promote the replication of vvDD (TK and VGF double-deleted VACV) and improve the survival in a malignant glioma rat model (Lun et al., 2009). Similarly, another study reported that rapamycin enhances MYXV susceptibility in racine glioma cells *in vitro* and prolongs survival when combined with MYXV in immunocompetent racine glioma rat models (Lun et al., 2010). Collectively, these findings suggest that autophagy modulation may serve as a viable target to enhance the efficacy of oncolytic poxviruses.

## 8 Conclusions and perspectives

In summary, this review summarizes current knowledge on the interplay between poxviruses and the intracellular membrane system, including autophagy and the endolysosome network. Given their cytoplasmic replication and extensive association with host membranes, poxviruses may interact with autophagy more intricately than currently appreciated. However, this field remains in its early stages, and further studies are needed to validate current observations and elucidate the underlying mechanisms. Many of the viral regulatory proteins encoded by poxviruses remain functionally uncharacterized. Among them may lie autophagy orchestrators. To unmask these elusive regulators, high-throughput overexpression or knockout screening are imperative. Moreover, the complexity of the poxvirus-host interaction is likely attributed to interspecies variation, the temporal and spatial regulation of viral protein expression and the variability of host cell types. In summary, we believe that further investigations into the interplay between poxviruses and the autophagy pathway will not only enhance our understanding of fundamental cellular processes, but also support the clinical applications of poxviruses.
